# Comparison of Different IOL Types in the Flanged IOL Fixation Technique

**DOI:** 10.1155/2020/8534028

**Published:** 2020-01-21

**Authors:** Yusaku Miura, Yosuke Harada, Yoshiaki Kiuchi

**Affiliations:** ^1^Department of Ophthalmology and Visual Science, Kochi Medical School, Kochi University, Kochi, Japan; ^2^Department of Ophthalmology and Visual Science, Graduate School of Biomedical Sciences, Hiroshima University, Hiroshima, Japan

## Abstract

**Purpose:**

To compare short-term clinical outcomes between two different intraocular lens (IOL) types in the flanged IOL fixation technique.

**Methods:**

This study was a retrospective case series and included the patients who underwent flanged IOL fixation between June 2017 and July 2018 at the Hiroshima University Hospital. Two different 3-piece IOLs (NX-70 and PN6A) were used. Recipients of NX-70 and PN6A IOLs were classed into groups 1 (15 eyes) and 2 (25 eyes), respectively. Patient characteristics, surgical results, and postoperative complications were analyzed. We excluded patients with a postoperative follow-up of <1 month.

**Results:**

The mean follow-up period was 13.3 ± 11.7 weeks. The postoperative best corrected visual acuity, in logarithm of the minimum angle of resolution (logMAR), was 0.10 ± 0.33 in group 1 and 0.26 ± 0.42 in group 2. The mean operation times for groups 1 and 2 were 11.2 ± 4.54 minutes and 7.00 ± 2.20 minutes, respectively (*p*=0.0024). Detachment of the IOL haptic from the optic during surgery occurred in four eyes in group 2 (16%), but did not occur in group 1. Iris capture of the optic was observed in 3 of the 13 eyes (23%) without a peripheral iridotomy in group 2. No peripheral iridotomies were performed on group-1 eyes, but iris capture did not occur in that group.

**Conclusions:**

There was a trend to fewer intraoperative and postoperative complications when using NX-70 IOLs. On the other hand, PN6A IOLs was easy to maneuver within the anterior chamber, and the operation time was shorter when using PN6A IOLs. Selection of optimal IOLs for flanged IOL fixation necessitates an understanding of their characteristics in terms of intraoperative and postoperative complications.

## 1. Introduction

Several surgical techniques for intraocular lens (IOL) implantation into aphakic eyes, in the absence of capsular support, have been reported, such as the use of anterior-chamber IOLs and iris-fixated IOLs and the transscleral suture-fixated IOL technique. However, these techniques can cause problems, including corneal endothelial loss, ocular hypertension, peripheral anterior synechiae, hyphema, and suture-associated complications [1–9]. Recently, sutureless scleral-fixated IOL techniques have been reported [10–13]. Compared with the suture-fixated IOL technique, sutureless techniques could reduce suture-associated complications such as suture-knot erosion; dislocated IOLs resulting from suture breakage; and suture-induced inflammation [1, 3–5, 8, 9]. A sutureless scleral-fixated IOL technique for IOL implantation into aphakic eyes that lack capsular support was first reported by Gabor and Pavlidis [10], with some subsequent modifications made by other surgeons [12, 13]. The novel flanged IOL fixation technique, reported by Yamane et al., comprises transconjunctival intrascleral IOL fixation with a double-needle technique using flanged haptics [14]. This technique is not reliant on scleral flaps, tunnels, sutures, fibrin glue, or exposure of the sclera by conjunctival incision. However, there is no dedicated IOL for this technique, and thus an existing IOL for intracapsular fixation must be used. Therefore, if the strength of the joint between a haptic and the optic is weak, serious complications, such as detachment of the haptic from the optic, may occur during surgery. In this study, we compared IOL characteristics, including the frequency of detached haptics and the strength of the joint, for two different types of IOLs for flanged IOL fixation.

## 2. Patients and Methods

This retrospective study adhered to the tenets of the Declaration of Helsinki and was approved by the Institutional Ethics Committee. It was granted a waiver of informed consent by the Institutional Ethics Committee because the data analyzed were deidentified records. The study included those patients who had undergone flanged IOL fixation in cases of aphakia, a subluxated lens, or a dislocated IOL. We used NX-70 (Santen, Osaka, Japan) and PN6A (Kowa, Tokyo, Japan) products for the IOL fixations. Both IOLs are three-piece acrylic lens with haptics made of polyvinylidene fluoride. NX-70 and PN6A have 7 mm and 6 mm optic diameters, respectively. The patients were divided into two groups, according to the type of IOL implanted: group 1 for the patients with the NX-70 and group 2 for those with the PN6A. All surgeries were conducted by a single surgeon (Y.M.) between June 2017 and July 2018 at the Hiroshima University hospital. We excluded patients with a postoperative follow-up of less than 1 month.

All patients underwent an ophthalmologic examination including best corrected visual acuity (BCVA), slit-lamp examination, and intraocular pressure (IOP) measurement at all preoperative and postoperative visits. IOL powers were calculated with the SRK/T formula using the IOL Master (Carl Zeiss Meditec, Jena, Germany). Refractive differences (postoperative spherical equivalent,refraction prediction) were measured 7 to 10 days after surgery. Operation time was measured as the time from IOL insertion to fixation in the center position. Complications associated with the surgical process were also compared between the two groups. Complications involving the haptics were considered intraoperative complications, specifically, detachment of a haptic from the optic or failure of externalization of the haptics (one or both haptics remained in the vitreous cavity after the needles were pulled out). Vitreous hemorrhage, ocular hypertension, hypotony, lens tilt, iris capture of the optic, exposure of a haptic, and cystoid macular edema were all recorded as postoperative complications. Ocular hypertension was defined as IOP > 25 mmHg. Hypotony was defined as IOP < 6 mmHg. Lens tilt was identified by slit lamp examination and confirmed by at least two expert ophthalmologists. Exposure of haptics was defined as the flange at the tip of the haptics being exposed on the conjunctiva. Cystoid macular edema was defined as intraretinal fluid visible on optic coherence tomography (Topcon 3D OCT-2000; Topcon, Tokyo, Japan).

**Figure 1:**
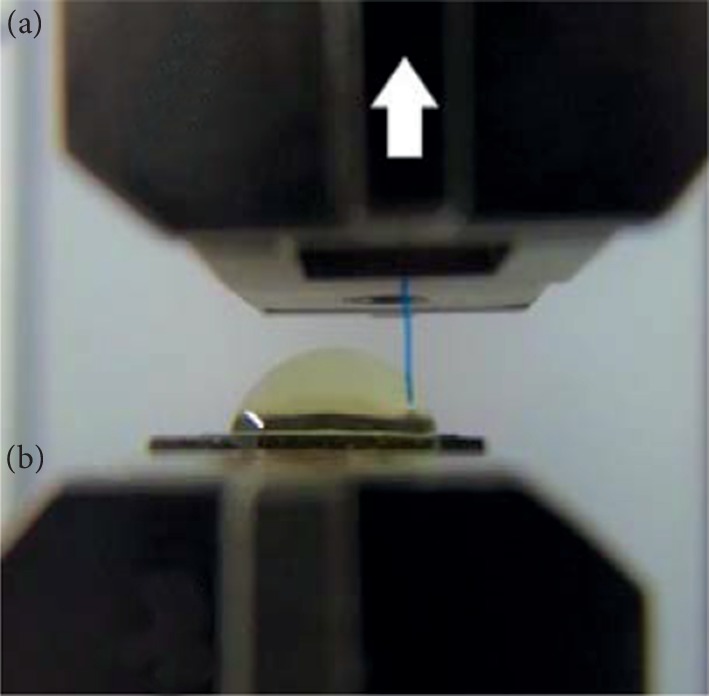
Testing the tensile strength of haptics. The tip of the haptic, grasped by pedestal A, was pulled horizontally with respect to the optic (arrow) at a speed of 3 mm/min, with the optic fixed on pedestal B. The load limit value was measured at the moment when a haptic was separated from the optic. The IOL power was 20 diopters in this test. Each test was repeated 15 times, and the mean values were calculated.

Although the IOLs used in this study are both three-piece acrylic lenses, they are manufactured by different companies. To determine the different characteristics of these lenses, beyond the optic diameter, the strength of the joints between haptics and optics in the NX-70 and PN6A was measured. For that measurement, 20-diopter NX-70 and PN6A lenses and an EZ-S-100N (Shimadzu Corporation, Kyoto, Japan) were used. With the optic fixed on the pedestal, the tip of the haptic was pulled at 3 mm/min in the horizontal direction with respect to the lens and we recorded the load limit value when the haptic was separated from the optic (Figure 1). Each test was repeated 15 times, and we calculated the mean values.

### 2.1. Surgical Technique

After peribulbar anesthesia using a sub-Tenon injection of 2% lidocaine, a 25-gauge or 23-gauge pars plana vitrectomy was performed. For patients with a subluxated lens, the lens was removed by phacoemulsification and aspiration. For patients with IOL dislocation, the IOL removal procedure was dependent on the IOL materials. If the IOL was an acryl lens, it was cut into two pieces and removed through a 3.0 mm corneoscleral incision. If the dislocated IOL was made of polymethyl methacrylate, it was removed through a 6.0 mm corneoscleral incision. The flanged IOL fixation followed the technique described in a previous report [14]. In short, a three-piece IOL was introduced into the anterior chamber with an injector, via a corneoscleral incision. The first sclerotomy was created at the 2- or 4 o'clock position and 2 mm from the limbus, using a 30-gauge thin-wall needle (TSK ultra-thin-wall needle; Tochigi Seiko, Tochigi, Japan). The leading haptic was grasped and inserted into the lumen of a needle using a pair of forceps. A second sclerotomy was created at the 8- or 10 o'clock position and 2 mm from the limbus with a 30-gauge thin-wall needle. The trailing haptic was grasped and inserted into the lumen of another needle. Then, the haptics were externalized by pulling out both needles. The ends of the haptics were then heated with an Accu-Temp cautery (Beaver Visitec Inc., Waltham, MA) to create flanges, and the flanges were pushed back and fixed intrasclerally. In 12 eyes of the group-2 patients, peripheral iridotomy was performed with a vitrectomy cutter after fixing the IOL in the center position. There were several cases in which a haptic separated from the optic during surgery. In such cases, the broken IOL was cut into two pieces and removed from the corneoscleral incision, and a new IOL was fixed intrasclerally. If a haptic turned back toward the inside of the eyeball, between pulling out the needle and creating a flange, the haptic was inserted into the lumen of the needle and externalized by pulling out the needle again.

### 2.2. Statistical Analysis

BCVA was converted to logarithm of the minimum angle of resolution (logMAR) units for the statistical analysis. The Wilcoxon rank-sum test was used to evaluate the group differences between continuous variables. The incidences of complications were compared between groups using the Fisher exact test. A *p* value of less than 0.05 was considered to be statistically significant. All data were entered into an Excel spreadsheet (Microsoft Corp, Redmond, WA) and analyzed using JMP® 11 (SAS Institute Inc., Cary, NC).

## 3. Results

Results from 40 eyes of 38 patients were included in this retrospective study. The mean follow-up was 13.3 ± 11.7 weeks. Group 1 comprised 15 eyes of 15 patients (8 males and 7 females) with a mean age of 70.8 ± 13.0 years. There were 4 aphakic eyes, 4 subluxated lenses, and 7 dislocated IOLs. Group 2 comprised 25 eyes of 23 patients (18 males and 5 females) with a mean age of 70.2 ± 13.6 years. There were 3 aphakic eyes, 14 subluxated lenses, and 8 dislocated IOLs (Table 1).

The mean preoperative BCVA (in logMAR) for group 1 was 0.40 ± 0.49, and for group 2, it was 0.64 ± 0.71 (*p*=0.23). The mean BCVA of about 1 month after surgery for group 1 was 0.10 ± 0.33, and for group 2, it was 0.26 ± 0.42 (*p*=0.26). The mean refractive differences were not statistically significant different between groups 1 and 2 (*p*=0.75). The mean operation times for groups 1 and 2 were 11.2 ± 4.54 minutes and 7.00 ± 2.20 minutes, respectively (*p*=0.0024) (Table 2).

**Figure 2:**
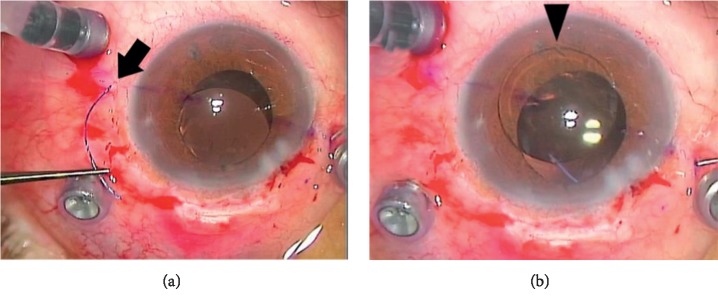
Intraoperative complications. (a) A haptic (arrow) detached from the optic. (b) The joint between the haptic and the optic (arrow head) from which the haptic was detached.

Table 3 collates the intraoperative and postoperative complications. In four eyes, the haptic detached from the optic as the needle was pulled out (Figure 2). Vitreous hemorrhage was observed in both groups; it resolved spontaneously within 1 week after the surgery in all three eyes. Ocular hypertension (>25 mmHg), which was seen in three eyes in group 2, was treated with IOP-lowering eye drops. These eye drops could be stopped within 1 month because of normalizing IOP. Ocular hypotension (<6 mmHg), which was observed in 10 eyes on the day following surgery, resolved spontaneously within 1 week. Lens tilt was observed in two eyes, but lens replacements were not conducted in those cases because the patients were satisfied with their postoperative BCVA. Iris capture of the optic was observed in three eyes in group 2, all of which recovered, through the use of a mydriatic agent in two of the eyes and by laser iridotomy in the third. The tip of the haptic was exposed above the conjunctiva in two eyes. These exposed haptics were pushed back with the tip of a forceps, without recurrence. Cystoid macular edema developed 2 months postoperatively in one eye and was cured within 1 month with nonsteroidal anti-inflammatory eye drops.

**Figure 3:**
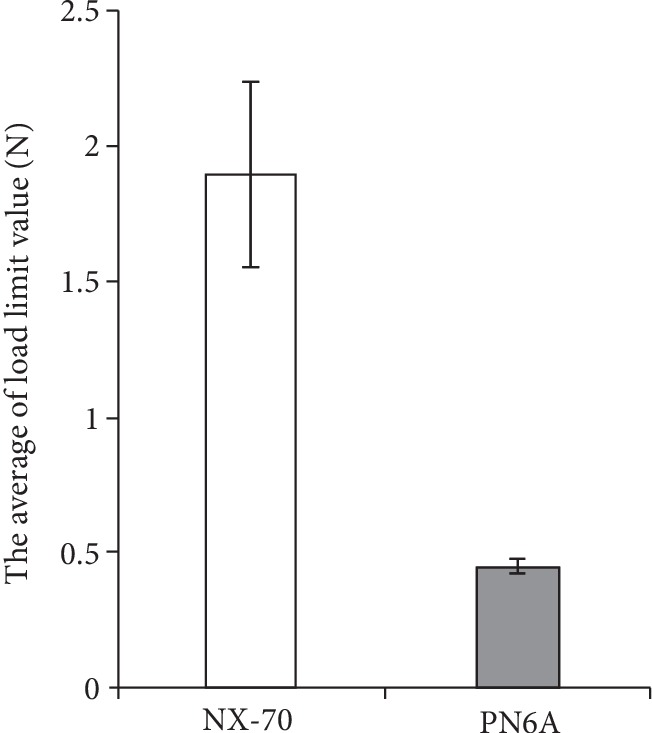
The results of the tensile-strength test. The joint strength of the NX-70 was significantly higher than that of the PN6A.

Figure 3 shows that the average load limit values for traction in the NX-70 and PN6A products were 1.90 ± 0.343 N and 0.449 ± 0.0302 N, respectively (*p* < 0.0001). That is, it was significantly lower in PN6A than in NX-70. In all cases, the haptics separated from the optics at the joint between a haptic and the optic. There was no case of the haptic itself breaking.

## 4. Discussion

The flanged IOL fixation procedure reported by Yamane et al. is an elegant new IOL fixation technique for patients without capsular support. Compared with other techniques, this technique is simple, less time-consuming, and minimally invasive [14]. This technique requires that the haptics of the IOL can form a flange when heated. Accordingly, most three-piece IOLs can be used for flanged IOL fixation [14]. Furthermore, an appropriate material for a haptic for this technique is polyvinylidene fluoride, which has sufficient flexibility to prevent breakage of haptics during insertion into the needles or pulling out of the needles. As such, NX-70 and PN6A are both suitable for this technique. Although there was no statistically significant difference in the incidence of intraoperative complications between the two groups, detachment of the haptic from the optic occurred in four eyes of group 2 (16%), whereas no detached haptics were observed in group 1 during surgery. Because the haptics of both IOLs are composed of polyvinylidene fluoride, which is unlikely to break, the strength of the joint between a haptic and its optic is a very important consideration when performing the flanged IOL fixation technique. If a haptic separates from the optic intraoperatively, the broken IOL should be removed and the IOL fixation procedure restarted, which can increase the chance of surgical complications such as the IOL dropping into the vitreous cavity, vitreous hemorrhage and retinal detachment, and corneal endothelial loss. Therefore, this intraoperative complication is very stressful for surgeons. Because the surgeon (Y.M.) performed the flanged IOL fixation for 18 eyes and got used to this procedure before the first experience of the detached haptic, we thought that the detached haptic did not occur simply due to the immaturity of this procedure. We showed that the strength of the joint between a haptic and the optic in the PN6A IOL is significantly lower than that in the NX-70, which presumably accounts for the high incidence of haptic detachments in group 2. During actual surgery, however, the load on the joint when grasping the haptic with forceps, inserting it into the 30-gauge thin-wall needle, or pulling out the needle is not only applied in the horizontal direction with respect to the lens, but the joint is also twisted. Furthermore, as the NX-70 and PN6A differ in haptic length (8.6 mm vs. 8.2 mm, respectively) as well as in optic diameter, the load on the joint during surgery cannot be simply compared. We need to know the strengths of the joints in different IOL products. If we use an IOL whose joint between the haptic and the optic is structurally vulnerable, we should handle a haptic carefully to avoid overloading the joints as much as possible. The most difficult procedure in the flanged IOL fixation technique is to insert a haptic into the lumen of a needle using a forceps. Beginners using this technique tend to overly twist and pull on haptics, placing excessive load on the joint. As a result, detachment of the haptic from the optic may occur. Therefore, we recommend that beginners should use NX-70 to avoid this intraoperative complication.

IOL tilt was observed in two eyes. To avoid IOL tilting, the first and second sclerotomies should be created with the same angle to the sclera, but that is not easy to do. Recently, needle guides have become commercially available. It is worth investigating whether a needle guide could prevent IOL tilting.

Since iris capture occurred in 3 of the first 13 eyes without a peripheral iridotomy in group 2, we performed a peripheral iridotomy in the following 12 eyes of group 2. Iris capture was neither observed in the 12 eyes with a peripheral iridotomy in group 2 nor in any of the group 1 eyes. A peripheral iridotomy may reduce the pressure difference between the anterior and posterior chambers, making iris capture less likely to occur in 6 mm optic IOL. In addition, the 7 mm optic IOL of group 1 may not need a peripheral iridotomy preventing for iris capture.

From our results, NX-70 IOLs seem to have an advantage in preventing IOL breakage and iris capture. However, PN6A IOLs have some other advantages. For example, the PN6A has a wide range of refraction degrees, from 7 to 30 diopters, whereas the NX-70 is only available for refraction degrees from 10 to 25 diopters. Additionally, PN6A IOLs have a smaller total length than NX-70 IOLs and are compact, so PN6A IOLs are easy to maneuver within the anterior chamber. For example, it is relatively easy with the PN6A to move an optic to the posterior chamber after inserting the leading haptic, in eyes with limited response to mydriatic eye drops, such as in patients with pseudoexfoliation, diabetes, or uveitis. Furthermore, because the tip of a leading haptic is located at the iridocorneal angle after inserting the IOL into the anterior chamber, it is more difficult to grasp the tip of a haptic with forceps for the NX-70 IOL than the PN6A. In fact, the operation time for the flanged IOL fixation was significantly shorter in group 2 than in group 1 (Table 2).

The limitations of our study are the small sample size, the nonrandomized nature of the study, and the short follow-up period. To clarify the different outcomes of procedures using these IOLs, a larger number of cases and longer follow-up period are needed. Furthermore, it is necessary to perform randomized studies to eliminate biases such as patient background.

In conclusion, although there was no statistically significant difference, there was a trend to fewer intraoperative and postoperative complications when using NX-70 IOLs. However, PN6A IOLs were easy to maneuver within the anterior chamber, and the operation time was shorter when using PN6A IOLs. We recommend that it is better for beginners using flanged IOL fixation technique to start using IOLs with strong joints such as NX-70 IOLs to avoid detachment of the haptic from the optic. Currently, there is no IOL designed specifically for the flanged IOL fixation technique. If the flanged IOL fixation is to be performed, we should first understand the advantages and the disadvantages of the existing types of IOLs.

## Figures and Tables

**Table 1:** Patient characteristics.

	Group 1 (NX-70)	Group 2 (PN6A)	*p*
No. of eyes	15	25	
Mean age (range), years	70.8 ± 13.0 (48–89)	70.2 ± 13.6 (43–89)	0.95
Sex			0.17
Male	8 (53.3%)	18 (78.3%)	
Female	7 (46.7%)	5 (21.7%)	
Eye			0.51
Right	9 (60.0%)	11 (44.0%)	
Left	6 (40.0%)	14 (56.0%)	
Diagnosis			
Aphakia	4 (26.7%)	3 (12.0%)	0.39
Subluxated lens	4 (26.7%)	14 (56.0%)	0.10
Dislocated intraocular lens	7 (46.6%)	8 (32.0%)	0.50
Axial length (range), mm	24.0 ± 1.51 (22.6–27.5)	25.1 ± 2.47 (22.3–31.9)	0.18

**Table 2:** Surgical results.

	Group 1	Group 2	*p*
Preoperative mean BCVA (logMAR)	0.40 ± 0.49	0.64 ± 0.71	0.23
Postoperative mean BCVA (logMAR)	0.10 ± 0.33	0.26 ± 0.42	0.26
Refractive difference	1.29 ± 1.91	0.93 ± 0.75	0.75
Mean operation time (minutes)	11.2 ± 4.54	7.00 ± 2.20	0.0024

**Table 3:** Complications.

	Group 1	Group 2	*p*
Intraoperative complications			
Detached haptic	0	4 (16.0%)	0.28
Failure of externalization of the haptics	0	1 (4.0%)	1.00
Postoperative complications			
Vitreous hemorrhage	2 (13.3%)	1 (4.0%)	0.54
Ocular hypertension	0	3 (12.0%)	0.27
Ocular hypotension	4 (26.7%)	6 (24.0%)	1.00
Lens tilt	1 (6.7%)	1 (4.0%)	1.00
Iris capture	0	3 (12.0%)	0.27
Exposed haptic	1 (6.7%)	1 (4.0%)	1.00
Cystoid macular edema	1 (6.7%)	0	0.38

## Data Availability

The data used to support the findings of this study are available from the corresponding author upon request.
